# Supplementary material for: A Flexible and Efficient Microfluidics Platform for the Characterization and Isolation of Novel Bacteriophages
Source: Appl Environ Microbiol. 2023 Jan 5;89(1):e01596-22. doi: 10.1128/aem.01596-22 (PMC9888219; doi:10.1128/aem.01596-22)
Supplement: Supplemental file 1 — Fig. S1 to S6. Download aem.01596-22-s0001.pdf, PDF file, 1.3 MB [file aem.01596-22-s0001.pdf]

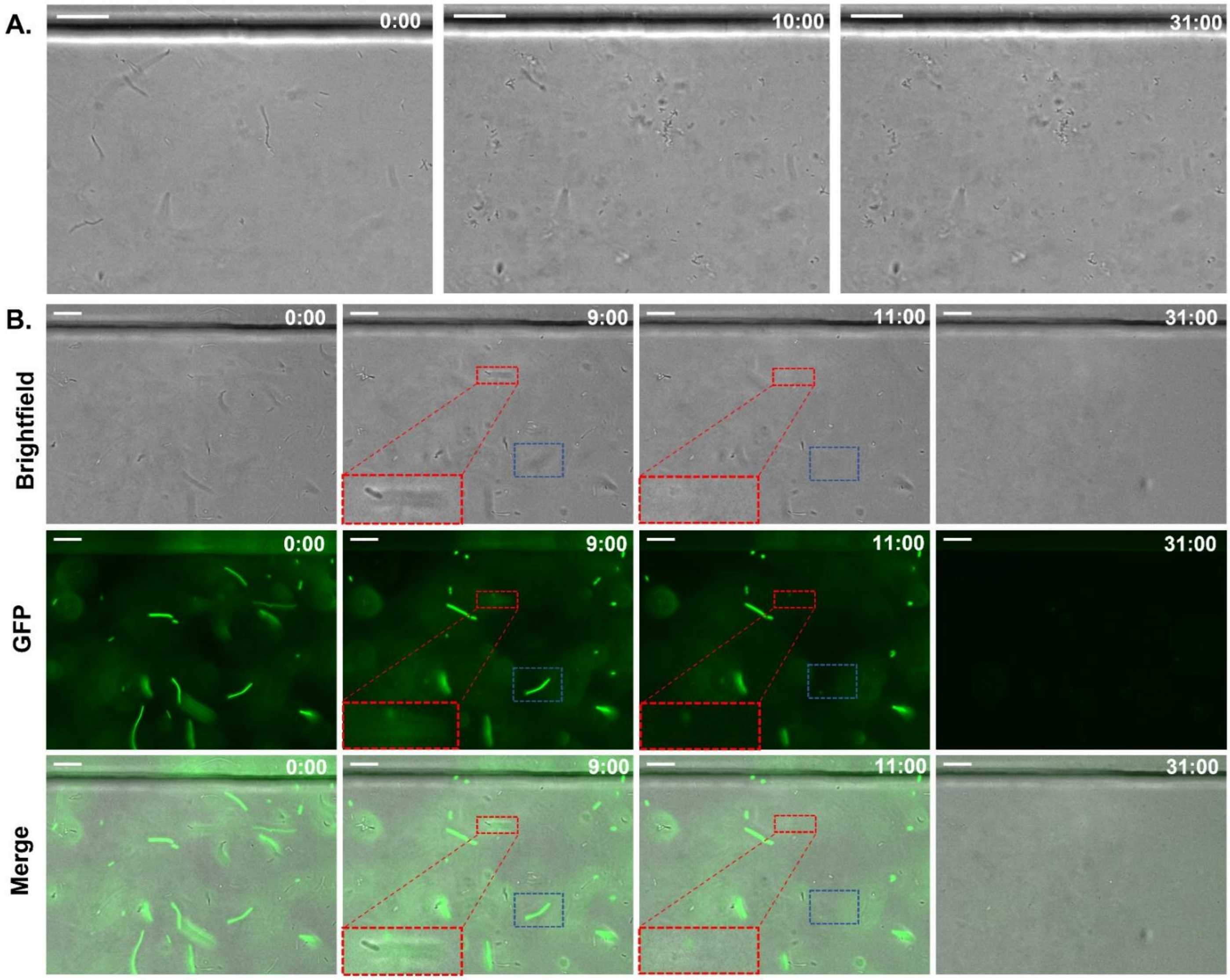

FIG S1. The effect of bacteriophage T4 on the growth of *E. coli* in brightfield and GFP in our system.

Images depicting *E. coli* over time in the platform in the absence (A) and presence (B) of T4 . The red and blue dashed boxes show examples of bacteria being lysed in both brightfield and GFP, with the red box showing a zoomed-in view in the bottom left. Time in hours is depicted in the top right corner. Scale bar is 15 µm. Note: Automated imaging may result in differences in focal plane between GFP and brightfield.

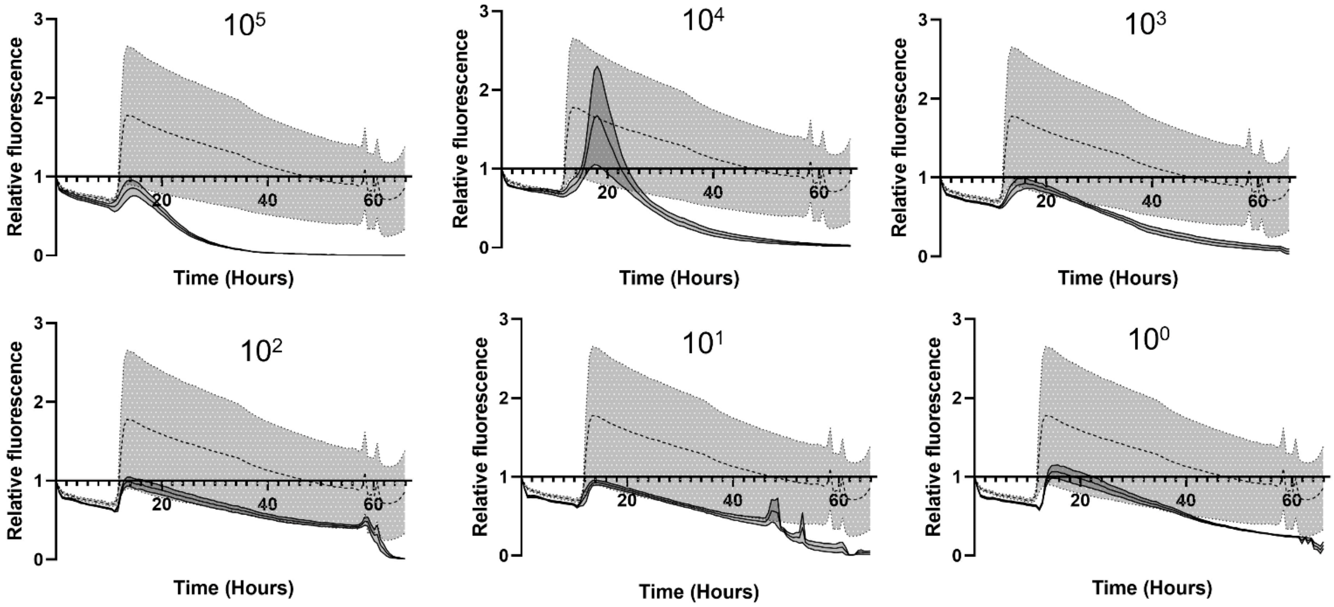

FIG S2. Relative fluorescence measurement of *E. coli* exposed by T4 at the lowest possible concentrations.

Various graphs depicting the relative fluorescent signal of GFP labeled *E. coli* over a period of 66 hours. The dotted line represents the control sample treated by phage buffer, while the solid line represents the sample that is treated by T4 phages. The number of phages used is depicted in each graph. Grey zones represent standard error of the mean (SEM).

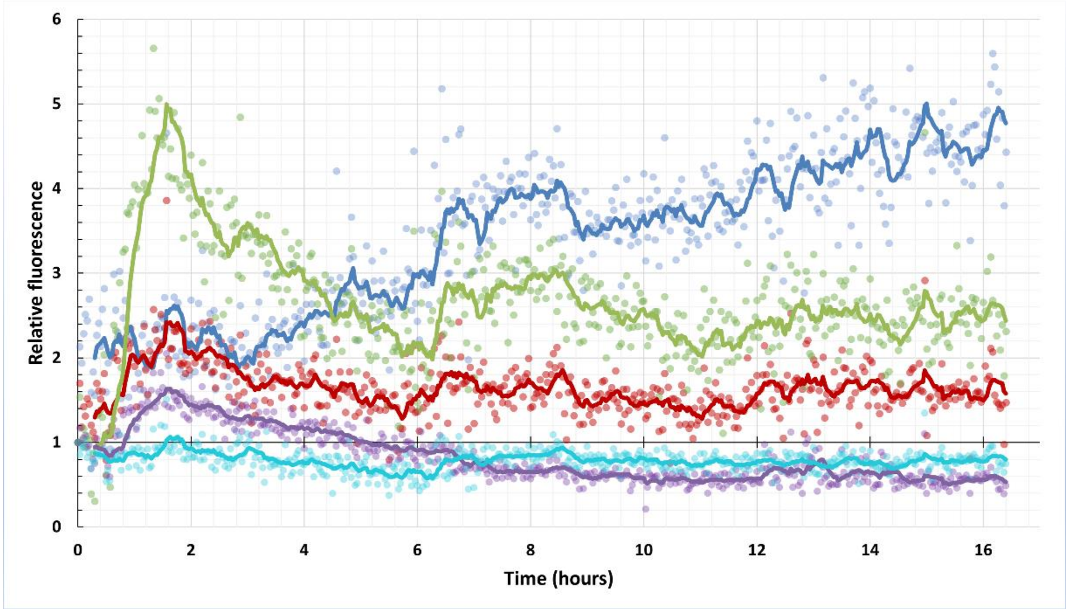

FIG S3. Plate reader data of Relative fluorescence of GFP labeled *E. coli* exposed to T4 at different MOI's. Graph depicting fluorescent signal relative to the first measurement of GFP labeled *E. coli* over a period of 16 hours. Treatment by T4 at different concentrations and the control are represented by different colors. The control is represented in blue. A MOI of 0.1, 1, 10 and 100 is represented by red, green, purple and teal respectively. The solid line represents the moving average (period: 10) of each respective treatment. Note: Increase in fluorescent signal before the 2 hour mark is presumably caused by a mechanical aberration.

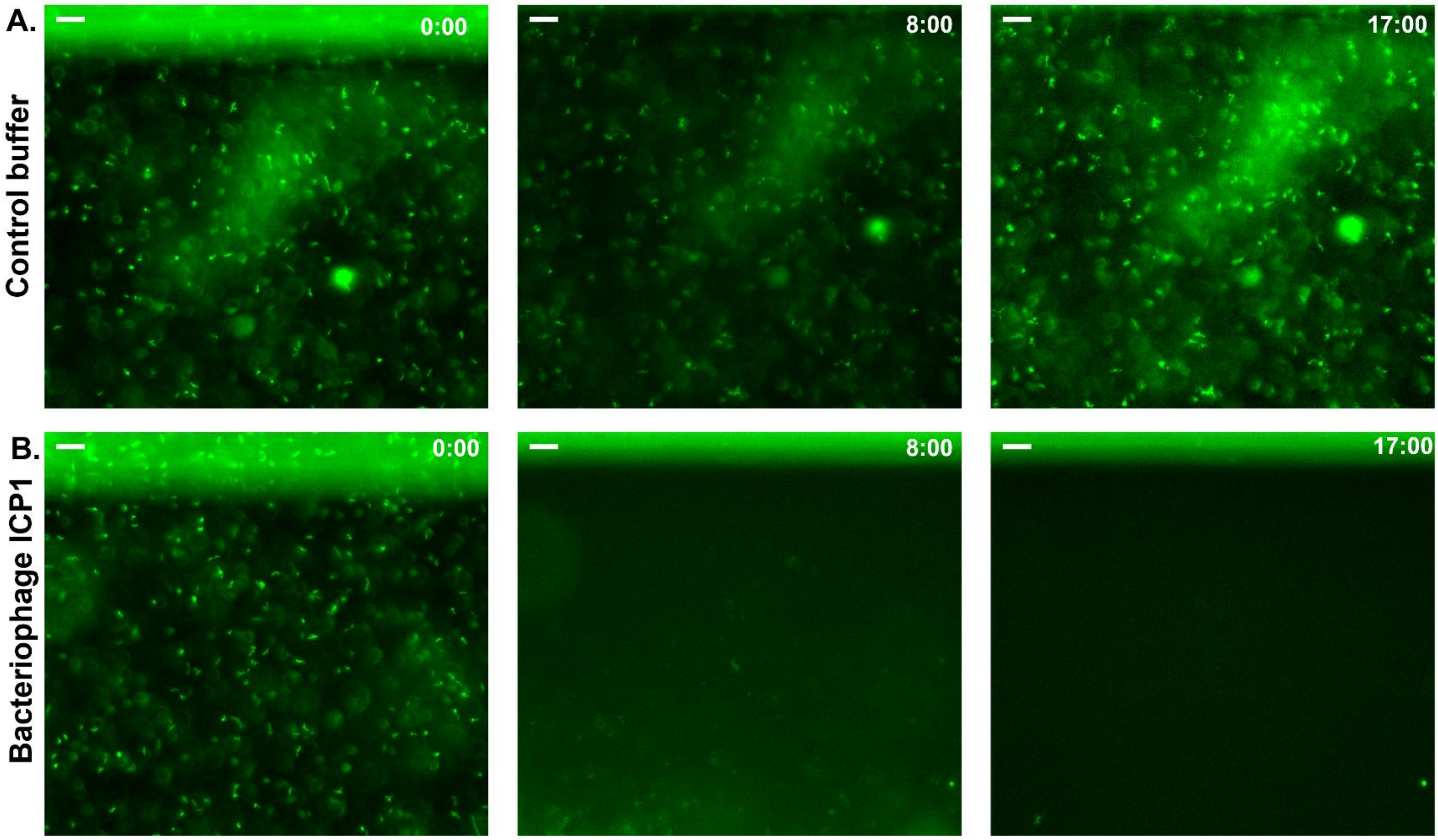

FIG S4. Images of GFP labeled *V. cholerae* exposed to control buffer or ICP1 (MOI: 10) at different timepoints. Images at specific timepoints depicting the effect of ICP1 exposure to *V. cholerae* cells over time. The control (A) and phage treated (B) conditions are depicted respectively from top to bottom. Time in hours is depicted in the top right corner. Scale bar is 15 µm.

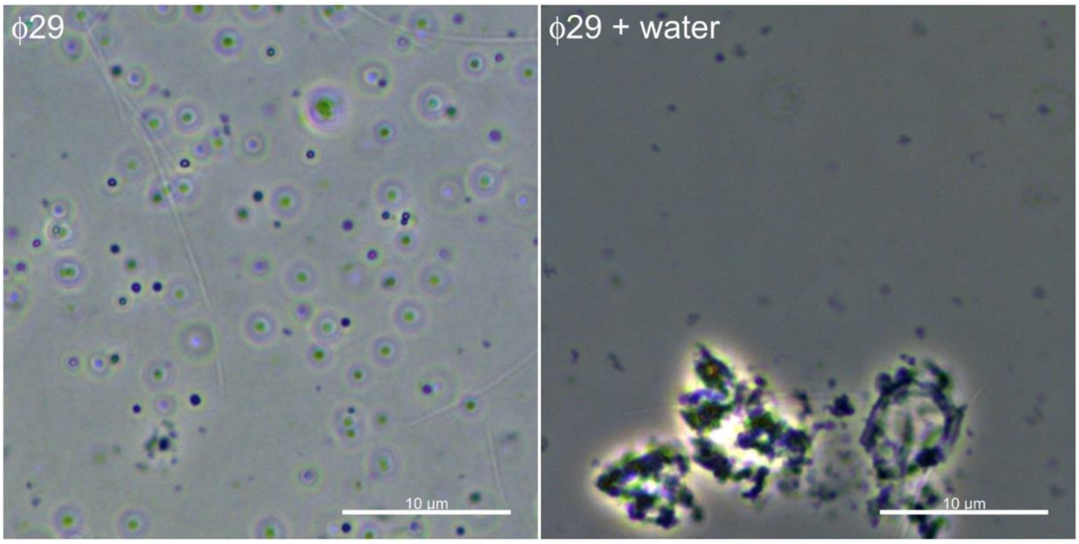

FIG S5. Images of *B. subtilis* cells after exposure to bacteriophage ϕ29 and to water.

Images depicting cell wall-deficient *B. subtilis* cells. These cells can be seen on the left after exposure to bacteriophage ϕ29. The image on the right showcases the same cells after the addition of water. Scale bar is 10 µm.

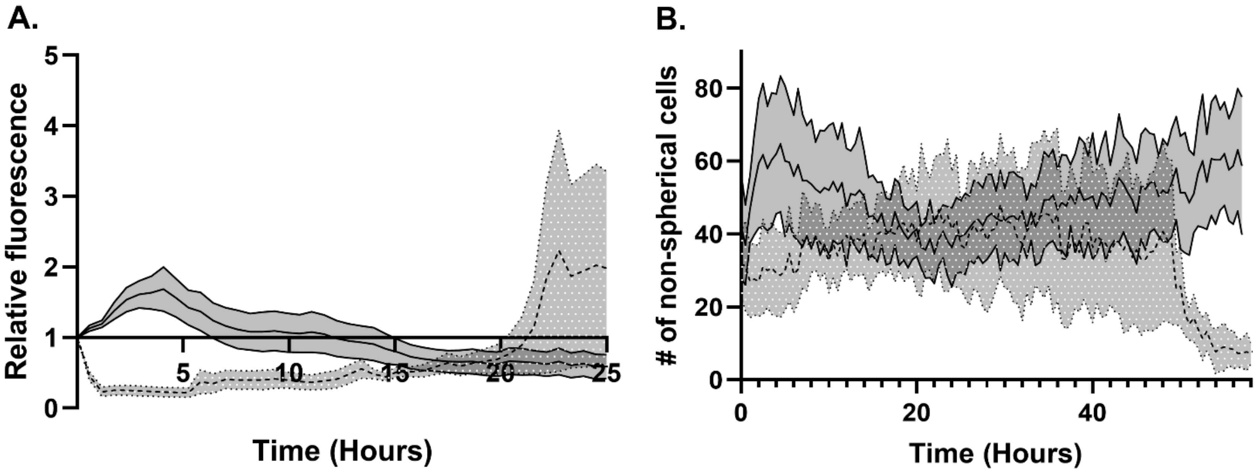

FIG S6. The effect of environmental water sample on *V. cholerae* and *B. subtilis*.

(A) Graph depicting fluorescent signal relative to the first measurement of GFP labeled *V. cholerae* over a period of 25 hours. Dotted line represents control sample that is exposed to phage buffer. Solid line represents *V. cholerae* that is exposed to the environmental water sample. Grey zones represent standard error of the mean (SEM). (B) Graph depicting the amount of non-spherical *B. subtilis* cells over a period of 66 hours. The dotted line represents the sample treated by phage buffer, while the solid line represents the sample that is treated by the environmental water sample. Grey zones represent standard error of the mean (SEM).
